# Cerebral Hyperperfusion Syndrome following Protected Carotid Artery Stenting

**DOI:** 10.1155/2013/207602

**Published:** 2013-07-11

**Authors:** Rainer Knur

**Affiliations:** Department of Cardiology and Angiology, Allgemeines Krankenhaus Viersen, Hoserkirchweg 63, 47147 Viersen, Germany

## Abstract

The cerebral hyperperfusion syndrome is a very rare complication after revascularization of the carotid artery and accompanied by postoperative or postinterventional hypertension in almost all patients. We report a case of a 77-year-old man who developed a complete aphasia and increased right-sided weakness following endovascular treatment of severe occlusive disease of the left internal carotid artery. We discuss the risk and management of cerebral hyperperfusion syndrome after carotid artery stenting.

## 1. Introduction

Neurological complications following carotid artery stenting (CAS) are usually ischemic in nature, due to embolization or occlusion of the carotid artery. However, in a small subset of patients, cerebral hyperperfusion causes postinterventional neurological dysfunction, characterized by ipsilateral headache, focal seizure activity, focal neurological deficit, and ipsilateral intracerebral edema or hemorrhage. A high clinical suspicion and early diagnosis will allow early initiation of therapy and preventing fatal brain swelling or bleeding in patients with peri- and postinterventional cerebral hyperperfusion syndrome (CHS).

## 2. Case Report

A 77-year-old man was referred for endovascular treatment after a transient ischemic attack with a right-sided facial and limb weakness. This episode occurred while the patient was undergoing medical treatment consisting of 100 mg acetylsalicylic acid and 75 mg clopidogrel 4 weeks after coronary stenting of the left anterior descending artery. The patient had a history of hyperlipidemia, hypertension, and familiar disposition with coronary heart disease. The neurological examination during the ischemic event revealed a mild right-sided hemiparesis. Brain CT and MRI showed no abnormalities. All hematological and biochemical tests were normal, with a normal platelet count and coagulation screen. 

**Figure 1:**
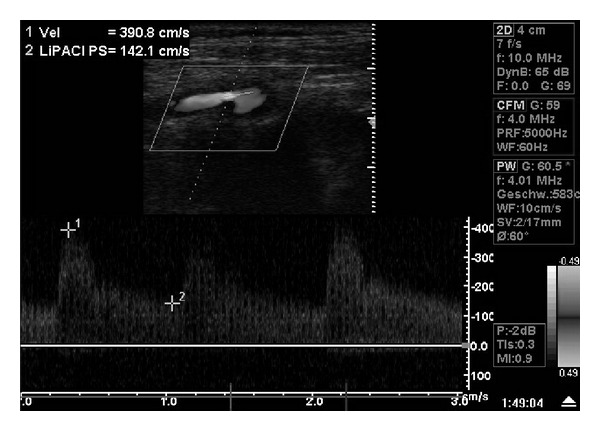
Color Doppler ultrasound of the left internal carotid artery (ICA). Severe stenosis of the left internal carotid artery with elevation of the peak systolic velocity at 3.9 m/s and an end diastolic velocity of 1.4 m/s.

**Figure 2:**
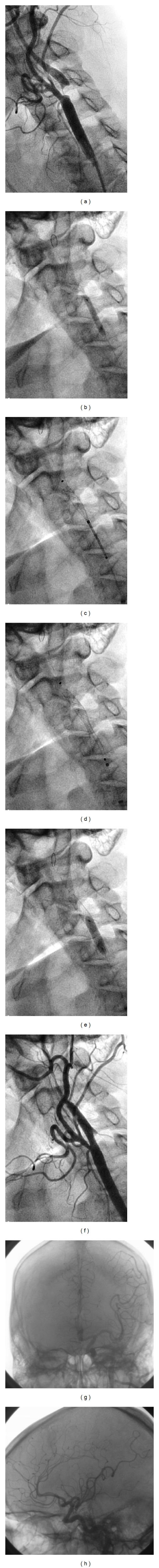
Carotid angiogram demonstrating carotid artery stenting (CAS) with distal filter protection in a 77-year-old symptomatic patient. (a) Preprocedural angiogram showing a high-grade stenosis of the left internal carotid artery. (b) After the filter is positioned distal to the lesion the stenosis is predilated with a 3 mm balloon. (c) The self-expanding stent is deployed. (d) Stent after deployment. (e) The stent is postdilated with a 5 mm balloon. (f) The final angiogram shows that the stented site is widely patent. (g), (h) Final angiogram of the intracranial vessels.

When assessed in our hospital, his blood pressure sometimes jumped up to 180/100 mm Hg. Therefore, the antihypertensive medication consisting of *β*-blocker, diuretic, and AT1-antagonist was intensified. Another neurological examination was normal. Color Doppler ultrasound showed a severe stenosis of the left internal carotid artery (ICA) with elevation of the peak systolic velocity at 3.9 m/s and an end diastolic velocity of 1.4 m/s (Figure 1). The patient got a loading dose of 500 mg ASS and 300 mg clopidogrel and underwent left carotid stenting the next day via a femoral approach under local anesthesia. The angiography confirmed 95% stenosis of the left ICA (Figure 2(a)). CAS was frictionless performed with distal filter protection, pre- and postdilation, and a self-expandable closed-cell design stent (Figure 2). The peri-interventional blood pressure varied between 140/85 to 160/95 mm Hg. The clinically stable patient was transferred to the intermediate care unit for monitoring.

20 minutes later the patient vomited, described ipsilateral headache, and became very anxious. He then developed a complete aphasia and increased right-sided weakness and became delirious. Blood pressure varied between 160/90 and 200/110 mm Hg. Immediately color Doppler ultrasound of the CCA and ICA revealed a visibly patent vessel. Brain edema and bleeding could be excluded by an urgent cranial CT. Followup within 24 hours with cranial MRI and angiography showed totally normal findings. The patient was transferred to the intensive care to control the hypertension and to monitor the vital parameters. Under intensified treatment of the blood pressure with a *β*-blocker, diuretic, AT1-, and Ca-antagonist, and temporary intravenous application of nitroglycerin and urapidil the neurological symptoms were totally regressed within few days. The patient was discharged after 10 days from the hospital. Follow-up examinations after 3 and 6 months were normal.

## 3. Discussion

In 1981, Sundt et al. [1] described a triad of complications that included atypical migrainous phenomena, transient focal seizure activity, and intracerebral hemorrhage after CEA and used the term cerebral hyperperfusion syndrome (CHS). The first report on CHS after CAS was published by Schoser et al. [2]. They described a 59-year-old woman with ipsilateral putaminal hemorrhage that was diagnosed on the 3rd day after CAS of a high-grade stenosis of the left ICA. Outcome in this case was not fatal. The patient recovered with a mild upper limb paresis. McCabe et al. [3] were the first to report the occurrence of fatal ICH soon after CAS. Only a few hours after the procedure, neurological symptoms occurred without any prodromata (severe headache, nausea, and seizures) postulated by Sundt et al. [1] to be an obligate component of CHS. CT of the brain revealed extensive ICH and the patient died 18 days later. Abou-Chebl et al. [4] reported a retrospective single-center study on 450 patients who had been treated with CAS. Three patients (0.67%) developed ICH after the intervention. Further reports on results and complications after CAS have been published [5]. Nearly all reports on CHS after carotid revascularizations in general and CAS in particular have in common patients who had high-grade stenoses in the treated vessel. 

CHS following surgical or endovascular treatment of severe carotid occlusive disease is thought to be the result of impaired cerebral autoregulation, hypertension, ischemia-reperfusion injury, oxygen-derived free radicals, baroreceptor-dysfunction, and intraprocedural ischemia [6]. Chronic cerebral hypoperfusion due to critical stenosis leads to production of vasodilatory substances. Autoregulatory failure results in the cerebral arterioles being maximally dilated over a long period of time, with subsequent loss of their ability to constrict when normal perfusion pressure is restored. The degree of microvascular dysautoregulation is proportional to the duration and severity of ischemia determined by the severity of ipsilateral stenosis and poor collateral flow. 

Hypertension plays an important role in the development of CHS. In the absence of cerebral autoregulation, cerebral blood flow is directly dependent on the systemic blood pressure. The restoration of normal blood flow to chronically underperfused brain can result in edema, capillary breakthrough, and perivascular and macroscopic hemorrhages aggravated by peri- and postinterventional hypertension [6, 7]. The risk factors for CHS after CAS are summarized in Table 1.

The classic clinical presentation includes ipsilateral headache, seizures or focal neurological deficit, and ipsilateral intracerebral edema or hemorrhage. The diagnosis can be made readily with color Doppler ultrasound of the carotid artery and especially with transcranial Doppler (TCD) of the middle cerebral artery [9]. An increase in peak blood flow velocity of >100% is predictive of postinterventional hyperperfusion. Diffusion weighted MRI or single photon emission computed tomography (SPECT) could also be performed for diagnosis [10]. Angiography normally shows normal findings. 

The prognosis of CHS depends on timely recognition of hyperperfusion and adequate treatment of hypertension before cerebral edema or hemorrhage develops. The prognosis following intracerebral bleeding is very poor, with mortality over 50% and significant morbidity of 80% in the survivors [4, 6]. The prognosis of CHS in patients without cerebral edema or hemorrhage is clearly better especially when they are identified and treated early. The most important aspects in preventing and treating this syndrome are early identification, careful monitoring, and control of blood pressure ideally in a high-dependency unit setting. In our special case, early diagnosis of CHS and immediate intensive medical treatment of blood pressure could prevent devastating cerebral edema or hemorrhage following CAS.

## 4. Conclusion

CHS, which is characterized by ipsilateral headache, hypertension, seizures, and focal neurological deficits, is a rare but devastating complication following carotid artery stenting. Hypertension is the most important risk factor. The diagnosis can be confirmed quickly by TCD, DWI, or SPECT. Especially peri- or postinterventional TCD monitoring should be available to identify patients with hyperperfusion who may benefit from intensive blood pressure management ideally in a specialized intensive care unit.

## Figures and Tables

**Table 1:** Risk factors for CHS [6–8].

Hypertension
High-grade stenosis with poor collateral flow
Decreased CVR
Increased peak flow velocity
Contralateral carotid occlusion or high-grade stenosis
Recent contralateral CAS or CEA within 3 months
Periprocedural ischemia
Presence of cerebral microangiopathy

CAS: carotid artery stenting, CEA: carotid endarterectomy, CHS: cerebral hypertension syndrome, and CVR: cerebrovascular reactivity.

## Abbreviations

CAS: Carotid artery stenting
CCA: Common carotid artery
CEA: Carotid endarterectomy
CHS: Cerebral hyperperfusion syndrome
CT: Computed tomography
CVR: Cerebrovascular reactivity
DWI: Diffusion-weighted imaging
ICA: Internal carotid artery
ICH: Intracerebral haemorrhage
MRI: Magnetic resonance imaging
SPECT: Single photon emission computed tomography
TCD: Transcranial Doppler.
